# Natural mortality estimates throughout the life history of the sea cucumber *Isostichopus Badionotus* (Holothuroidea: Aspidochirotida)

**DOI:** 10.7717/peerj.5235

**Published:** 2018-07-18

**Authors:** Salvador Romero-Gallardo, Iván Velázquez-Abunader, Jorge Alberto López-Rocha, Eduardo Garza-Gisholt

**Affiliations:** 1 Departamento de Recursos del Mar, Centro de Investigacion y de Estudios Avanzados del Instituto Politecnico Nacional, Merida, Yucatan, Mexico; 2 Laboratorio de Análisis Espacial de Zonas Costeras, Unidad Multidisciplinaria de Docencia e Investigación, Facultad de Ciencias, Universidad Nacional Autónoma de Mexico, Sisal, Yucatan, Mexico

**Keywords:** Gnomonic intervals, Natural mortality-at-age, *Isostichopus badionotus*, Sea cucumber, Stock assessment, Fishery, Sea cucumber

## Abstract

The Natural Mortality coefficient (*M*) is a key parameter for stock assessments. The need to establish age-specific natural mortality coefficients is widely recognized because *M* decreases rapidly over the early stages of the life cycle until it reaches a stable *M* value around the age-at-maturity. The aim of this study was to estimate *M* during the life cycle of the sea cucumber *Isostichopus badionotus*, a species under heavy fishing exploitation in the Caribbean Sea and the Gulf of Mexico. Coefficients *M* at age were estimated using two models: The Gnomonic Interval Model (GIM) and the Chen & Watanabe model. Two different scenarios were simulated considering early and late age-at-maturity. Estimated *M* values using the GIM model for the early maturity scenario were 2.15 to 2.35 year^−1^ (interquartile range) for the juvenile stage and 0.39 to 0.43 year^−1^ for the adult stage; for the late maturity scenario were 0.65 to 0.71 year^−1^ for the juvenile stage and 0.68 to 0.74 year^−1^ for the adult stage. The Chen & Watanabe model *M* estimates for juvenile stage were between 0.85 and 2.23 year^−1^ and 0.39 and 2.23 year^−1^ for the early and late maturity scenarios respectively; for adult stage were between 0.97 and 0.21 year^−1^ and 0.62 and 0.43 year^−1^ respectively. The GIM estimated high natural mortality rates during larval stages. These estimates provided a higher level of certainty for the population models to more effectively manage a fishery and improve stock assessments.

## Introduction

The high demand for sea cucumbers (Echinodermata: Holothuroidea) in the Asian market has led to expansion of their fisheries in countries where these organisms are not traditionally consumed (Toral-Granda, Lovatelli & Vasconcellos, 2008; Purcell et al., 2013; Eriksson et al., 2015). Additionally, the biological characteristics of holothurians, such as their low motility, low recruitment rate, high longevity and density-dependent reproductive success result in high vulnerability and a high risk of overexploitation of this resource (Bruckner, 2005; Purcell et al., 2010).

Studies of the population dynamics of sea cucumbers have focused on species with a long history of exploitation (Anderson et al., 2011; Purcell et al., 2013), such as *Apostichopus japonicus* (Selenka, 1867; Yang, Hamel & Mercier, 2015), *Cucumaria frondosa* (Semper, 1868; Hamel & Mercier, 2008) and *Isostichopus fuscus* in Mexico (Herrero-Pérezrul et al., 1999; Reyes-Bonilla & Herrero-Pérezrul, 2003; Glockner-Fagetti, Calderon-Aguilera & Herrero-Pérezrul, 2016). In most Latin American countries, however, there is limited information about the status and population dynamics of more recently exploited holothuroids (Toral-Granda, Lovatelli & Vasconcellos, 2008). It is therefore important to obtain precise estimates of their population biology parameters, based on a knowledge of their life history. A critical parameter of interest is the natural mortality coefficient (*M*) which provides an indicator of the rate of death in a population.

Estimating realistic values of *M* is a real challenge because different factors such as depredation, competition, diseases, among others must be considered. That is why in the stock assessment, estimates of *M* are used through empirical models, where their estimates are at the expense of being validated with observations in the field.

### A natural mortality coefficient in sea cucumbers

The natural mortality coefficient (*M*) is an important input parameter in age-structured models or other stock assessment models that actually uses the *M* as their primary input parameter; also as sustainability indicators, optimal exploitation rates and other biological reference points (Siegfried & Sansó, 2009; Brodziak et al., 2011). The *M* coefficient includes the result of all possible causes of natural death other than fishing, and direct estimates can be calculated for exploited and unexploited stocks (McPherson, García-Rubies & Gordoa, 2000; Bacheler et al., 2009). The *M* coefficient can be calculated using tag-recapture and telemetry methods (Pollock, Jiang & Hightower, 2004; Polacheck et al., 2006; Hewitt et al., 2007; Bacheler et al., 2009), visual population census data, catch-at-age models (McPherson, García-Rubies & Gordoa, 2000; Giménez-Hurtado, Arreguín-Sánchez & Lluch-Cota, 2009), and mortality by predation using Multispecific Virtual Population Analysis (MVPA) (Sparholt, 1990). Other methods such as integral evaluation models or those derived from ecosystem models (Walters, Christensen & Pauly, 1997; Arreguín-Sánchez, 2000) can also be applied when large data sets are available. Given the complexity of direct methods, the use of indirect methods based on life span estimates can provide an adequate solution to infer values of *M* while validating these values through direct methods (Then et al., 2014).

The natural mortality coefficient *M* may vary during an organism’s life cycle (Walters, Christensen & Pauly, 1997; Jennings, Kaiser & Reynolds, 2009); especially in species with high fecundity, higher *M* values are obtained during early life history stages, and these values are gradually reduced to attain an equilibrium value once the organism reaches the adult phase (Caddy, 1996). Natural mortality coefficients can also decrease exponentially until an organism reaches the maximum age (Chen & Watanabe, 1989; Caddy, 1996; Jennings, Kaiser & Reynolds, 2009). The use of a constant *M* coefficient to evaluate a given population/stock, however, results from the complexity and uncertainty in the estimate of this parameter, as it assumes that all the exploited age groups have the same probability of death (Hewitt & Hoenig, 2005). Therefore, at present, it is necessary to make this assumption when size- or age-specific estimates of *M* for a particular resource are unavailable (Caddy, 1996; Andrews & Mangel, 2012).

Thus, for some sea cucumber species, there are estimates of *M* based mainly on the assumption that *M* remains constant during the life cycle of the species (Ebert, 1978; Herrero-Pérezrul et al., 1999; De la Fuente-Betancourt et al., 2001; Reyes-Bonilla & Herrero-Pérezrul, 2003). Other studies commonly estimate *M* using empirical equations based on a consideration of growth parameters (Pauly, 1980). These empirical equations use as input information such to the longevity of the organisms (Hoenig, 1983), age-at-maturity (Rikhter & Efanov, 1976), growth (only for the von Bertalanffy model), and mortality as a function of temperature (Beverton & Holt, 1959; Pauly, 1980).

### The sea cucumber *Isostichopus badionotus*

*Isostichopus badionotus* (Selenka, 1867), also known as the four-sided sea cucumber, can grow to a maximum length of 45 cm (Toral-Granda, Lovatelli & Vasconcellos, 2008). There is at present very limited, information on the status of sea cucumber fisheries in the Caribbean region, only the studies of Buitrago & Boada (1996), De la Fuente-Betancourt et al. (2001), Alfonso et al. (2004) and López-Rocha, Cisneros-Reyes & Arreguín-Sánchez (2012), have addressed in some way the subject of their fisheries assessment. Yet intensive exploitation of *Isostichopus* species is carried out in several areas of the Caribbean Sea and the Gulf of Mexico. As a result, these populations are declining due to overexploitation and a lack of harvest regulations (Toral-Granda, Lovatelli & Vasconcellos, 2008). Only few countries (e.g., Cuba and Mexico) have regulated fishing activities by implementing catch quotas, a minimum capture size and closed fishing season (Alfonso et al., 2004; DOF, 2015). In some countries (e.g., Colombia), a total ban has been imposed on sea cucumber fishing (Lovatelli & Sarkis, 2011; Rodríguez-Forero & Agudelo-Martínez, 2017).

Different studies on biological and ecological aspects of *Isostichopus badionotus* populations have been conducted, these studies include aspects dealing with their geographic distribution (Guzmán & Guevara, 2002; Hasbún & Lawrence, 2002; Zetina-Moguel et al., 2003; Rodríguez et al., 2007; Tagliafico, Rangel & Rago, 2011; López-Rocha, Cisneros-Reyes & Arreguín-Sánchez, 2012; Hernández-Flores et al., 2015), reproduction, feeding and growth (Hammond, 1982; Sambrano, Díaz & Conde, 1990; Guzmán, Guevara & Hernández, 2003; Foglietta et al., 2004; Vergara & Rodríguez, 2015; Poot-Salazar, Hernández-Flores & Ardisson, 2015), and more recently immunology and aquaculture (Klanian, 2013; Zacarías-Soto et al., 2013; Zacarias-Soto & Olvera-Novoa, 2015; Martínez-Milián & Olvera-Novoa, 2016). There has been, however, no prior assessment of natural mortality coefficients for this species to date, and no such values have been reported in databases such as http://www.sealifebase.org (Palomares & Pauly, 2010).

Therefore, the aim of the present study was to estimate, for the first time, the natural mortality coefficient, *M,* using two indirect methods that take the age of the organism into consideration. These are a model based on gnomonic intervals (Caddy, 1996) and a model of the natural mortality coefficient at age developed by Chen & Watanabe (1989).

**Table 1:** Input data for Gnomonic (GIM) (Caddy, 1996) and Chen & Watanabe (1989) models.

Stage/parameter	Early maturity (1.5 years)	Late maturity (5 years)
Early auricularia (EA)	2 days	2 days
Medium auricularia (MA)	3 days	3 days
Late auricularia (LA)	6 days	6 days
Doliolaria-pentactula (D+P)	11 days	11 days
Juvenile (J)	537 days	1,803 days
Adult (A)	3,091 days	1,847 days
Fecundity (*f*)	13,500–5,062,490 ovules	13,500–5,062,490 ovules
*k*	0.2 to 0.7 year^−1^	0.2 to 0.7 year^−1^
*t*_0_	−0.8 to −0.4 year	−0.8 to −0.4 year
Age at maturity	1.5 years (547.5 days)	5 years (1,825 days)

**Notes.**

*k* is the body growth coefficient from von Bertalanffy model, *t*_0_ is the theoretical age when the length is equal to zero from von Bertalanffy model.

## Materials and Methods

### The gnomonic intervals model

The natural mortality coefficient (*M)* estimate obtained through application of the gnomonic interval model (Caddy, 1996) assumes that *M* decreases rapidly with age. Additionally, the life cycle of the species may be divided into intervals such that their duration increases as a function of age (Caddy, 1996). This method that subdivides the life cycle is referred to as “gnomonic”. A constant probability of death is assumed during each gnomonic interval. Estimation of the *M* parameter requires definition of the number of development stages considered during the life cycle of the species, the duration of the first stage, which corresponds to the first gnomonic time interval, the mean fecundity (annually) and the duration of each subsequent stage of the life cycle.

For implementation of this model, six different stages of development were considered during the life cycle of *I. badionotus*: four larval stages in addition to one juvenile stage and the adult stage. The first four stages comprised the early auricularia with a duration of two days, a medium auricularia stage lasting three days, and finally a late auricularia and doliolaria-pentactula stage, lasting six and 11 days, respectively (Zacarías-Soto et al., 2013) (Table 1). The last two larval stages were combined to allow convergence in the gnomonic calculation, since the model assumes a series of intervals of increasing duration (Caddy, 1996). The juvenile and adult stages were defined according to the age-at-maturity. It is important to know the effect of the age-at-maturity (*t*_*m*_) in the estimation of mortality. Two different scenarios varying the age-at-maturity of 1.5 and five years (early and late age-at-maturity respectively) were compared (Table 1). Poot-Salazar (2014) estimated the length-at-maturity based on microscopic observations of the gonads of ∼1,000 individuals caught in the coasts of Yucatan, Mexico in which the highest concentration of mature organisms (25% and 75% percentiles of dorsal length) was found between 20 and 30 cm of dorsal length (equivalent to 18.5 and 32.2 cm Le respectively) corresponding to ∼1.5 and ∼5 years of age (Poot-Salazar, Hernández-Flores & Ardisson, 2015). For this reason, we decided to use both scenarios to avoid discarding the variability in the size and age-at-maturity of this species. Therefore, the age-at-maturity of 1.5 years was defined as early maturity (scenario 1) and age-at-maturity of five years as late maturity (scenario 2) (Table 1).

The age-at-maturity was calculated from the length-at-maturity (23 cm dorsal length equivalent to estimate length (Le) = 21.9 cm) reported for this species by (Poot-Salazar, Hernández-Flores & Ardisson, 2015). The inverse of the von Bertalanffy growth model was used to calculate the age at which the species reaches 23 cm in dorsal length (Le = 21.9 cm). Two sets of parameters of the seasonal von Bertalanffy growth model reported by (Poot-Salazar, Hernández-Flores & Ardisson, 2015) were used. The growth parameters were estimated for 2,347 sea cucumbers collected by previous authors in three zones in Yucatan coast through the analysis of modal progression. Electronic Length Frequency Analysis ELEFAN-1 and Projection Matrix PROJMAT were methods used to estimate growth parameters (Poot-Salazar, Hernández-Flores & Ardisson, 2015).

The duration of the first larval stage (*t*_1_) corresponded to the first interval of gnomonic time (Δ_1_), then Δ_1_ = *t*_1_. That of the second gnomonic interval was obtained as Δ_2_ = *α* × *t*_2−1_, where *α* is a proportionality constant (Caddy, 1996). This constant minimizes the residuals until the sum of the duration of all the gnomonic intervals equals the longevity of the species (*t*_*n*_) using the Newton algorithm (Neter et al., 1996). The successive gnomonic time intervals were calculated as Δ_*i*_ = (*α* × *t*_*i*__−1_) + *t*_*i*__−1_ where i ≥ 3 to the *n –th* gnomonic interval, and *n* is the number of intervals. The natural mortality coefficient in each gnomonic interval, *M*_*i*_, was estimated from the following expression:

(1) \begin{eqnarray*}{M}_{i}= \frac{G}{{\theta }_{i}-{\theta }_{i-1}} \end{eqnarray*}

where *G* is the constant death probability for each gnomonic interval determined by numerical methods using Newton algorithm (Neter et al., 1996). The value of *G* is the product of *M*_*i*_ by Δ_*i*_ and *G* parameter which allows to calibrate the duration time of the gnomonic intervals. To date, there is no published evidence to determine if *G* parameter can be considered as an attribute of the species studied. The number of surviving individuals in the last interval is *N*_*i*_ = 2 (Caddy, 1996), *θ* is the difference of the annual proportional duration of each interval, calculated as *θ*_*i*_ = (Δ_*i*_∕*t*_*n*_)∕365, and where *t*_*n*_ is the total longevity of the species in days (Martínez-Aguilar et al., 2010). Because the actual longevity of this species has not been proven, we tested simulating *M* using three longevity (10, 20 and 30 years) in the present study and found that after 10 years the values of *M* did not change significantly (*P* > 0.05). Therefore, the results correspond to a longevity of 3,650 days (∼10 years); this longevity is similar to that supported by a maximum predicted age for this species (based on length-age and weight-relationships) from 10 to 12 years (Poot-Salazar, Hernández-Flores & Ardisson, 2015).

The number of survivors (*N*_*i*_) at the beginning of each interval (*i –th)* is equal to the number of survivors from the previous interval, except for the first interval for which it is assumed that the total number of individuals at the beginning of the interval equals the individual average fecundity per year of the species ( $\bar {f}$), determined from the following equations:

(2) \begin{eqnarray*}{N}_{i}& =\bar {f}{e}^{-{M}_{i}{\theta }_{i}}\quad \mathrm{for}\;i=1\end{eqnarray*}

(3) \begin{eqnarray*}{N}_{i}& ={N}_{i-1}{e}^{-{M}_{i}{\theta }_{i}}\quad \mathrm{for}\;i\gt 1\end{eqnarray*}

Variability in *M* was evaluated considering that the main source of uncertainty in the estimates is contributed by *f*. A total of 1,000 repeated samples of *f* were simulated to determine the uncertainty of the *M*_*i*_ estimates; then *M*_*i*_ estimates were obtained from 1,000 simulations per *i* gnomonic interval. A uniform, non-informative distribution (U) of *f* was assumed as follows:

(4) \begin{eqnarray*}f\approx \mathrm{U}({f}_{min}.{f}_{max})\end{eqnarray*}

where *f*_*min*_ and *f*_*max*_ are the minimum and maximum fecundity values reported, respectively (Martínez-Aguilar, Arreguín-Sánchez & Morales-Bojórquez, 2005).

For *I. badionotus*, the *f* estimate is uncertain and to date no specific estimate of the annual fecundity is available. Direct counts of the number of eggs released per spawning in captivity were used for this calculation, which yielded an average of 200,000, and a range from 13,500 to 1,066,000 (minimum and maximum) eggs per spawn per female (*n* = 83 spawnings) (Zacarías-Soto et al., 2013). Additionally, the estimated fecundity using a volumetric method from gonadal microscopic analysis yielded a range between 62,496 and 5,062,490 ova with a mean of 1,417.330 ±151,186 (standard deviation, SD) ova (*n* = 175 specimens) (Palazon, 2001). This was based on the assumption that the number of hatched larvae is equivalent to the individual fecundity of the species (Caddy, 1996), and includes all estimates until capture, thus representing the total lifespan variability.

Estimates of *M* were obtained with the Gnomonic-Interval Natural-Mortality Method (GIM) routine for Excel (Morales-Bojórquez et al., 2003), which allows the incorporation of observed data during the specific duration of each developmental stage of the species to calibrate estimates of the *M* vector (Martínez-Aguilar et al., 2010).

### The Chen & Watanabe model

This model is derived from the relationship described by Chen & Watanabe (1989), in which there is a qualitative correspondence between the growth stage and *M*. Therefore, *M* can be calculated at any age (*t*). It is necessary to know the body growth coefficient (*k)* and the theoretical age when the length is equal to zero (*t*_0_) of the von Bertalanffy growth model (Von Bertalanffy, 1938). For this model, *M* is assumed to be inversely proportional to growth (*k*), using age-at-maturity (*t*_*m*_) explicitly. The *M*_*t*_ estimate was derived using the following equations:

(5) \begin{eqnarray*}{M}_{t}& = \frac{k}{ \left( 1-{e}^{-k \left( t-{t}_{0} \right) } \right) } \quad \mathrm{for}\; t\leq {t}_{m}\end{eqnarray*}

(6) \begin{eqnarray*}{M}_{t}& = \frac{k}{{\gamma }_{0}} +{\gamma }_{1} \left( t-{t}_{m} \right) +{\gamma }_{2}(t-{t}_{m})^{2} \quad \mathrm{for}\;t\gt {t}_{m}\end{eqnarray*}

where *M*_*t*_ is the average natural mortality at age *t*, *γ*_0_, *γ*_1_ and *γ*_2_ are parameters of the model calculated based on the continuity of growth to the age *t*_*m*_ as follows:

(7) \begin{eqnarray*}{\gamma }_{0}& =1-{e}^{-k({t}_{m}-{t}_{0})}\end{eqnarray*}

(8) \begin{eqnarray*}{\gamma }_{1}& =k{e}^{-k({t}_{m}-{t}_{0})}\end{eqnarray*}

(9) \begin{eqnarray*}{\gamma }_{2}& =-0.5{k}^{2{e}^{(-k({t}_{m}-{t}_{0}))}}\end{eqnarray*}

As in the GIM method above, two different scenarios of age-at-maturity ( *t*_*m*_) were considered (early and late age-at-maturity).

The number of survivors to the age *N*_*t*_ were estimated from:

(10) \begin{eqnarray*}{N}_{t}& ={N}_{0} \frac{1-{e}^{k{t}_{0}}}{1-{e}^{-k \left( t-{t}_{0} \right) }} {e}^{-kt}\mathrm{for}t\leq {t}_{m}\end{eqnarray*}

(11) \begin{eqnarray*}{N}_{t}& =N \left( {t}_{m} \right) \left[ \frac{2{\gamma }_{2}(t-{t}_{m})({\gamma }_{1}-\sqrt{{\gamma }_{1}^{2}-4{\gamma }_{0}{\gamma }_{2}})}{2{\gamma }_{1}(t-{t}_{m})({\gamma }_{1}-\sqrt{{\gamma }_{1}^{2}-4{\gamma }_{0}{\gamma }_{1}})} \right] \frac{k}{\sqrt{{\gamma }_{1}^{2}-4{\gamma }_{0}{\gamma }_{1}}} \quad \mathrm{for}\;t\gt {t}_{m}\end{eqnarray*}

where:

(12) \begin{eqnarray*}N \left( {t}_{m} \right) ={N}_{0} \frac{1-{e}^{k{t}_{0}}}{1-{e}^{-k({t}_{m}-{t}_{0})}} {e}^{-k{t}_{m}}\end{eqnarray*}

and *N*_0_ is the estimated survival for time *t* − 1 or the fecundity of the species when *t* = 1 (Chen & Watanabe, 1989).

It is important to characterize the variability associated with these estimates, because the use of a single, discrete estimate without error measures underestimates the uncertainty associated with the results for future evaluations (Brodziak et al., 2011). In this study, error measures were added to the (Chen & Watanabe, 1989) model, assuming that the main source of uncertainty is generated by the parameters *k* and *t*_0_.

The present study was based on the minimum and maximum values of the parameters *k* and *t*_0_ of the von Bertalanffy model reported for this species in different locations of the Gulf of Mexico (Poot-Salazar, Hernández-Flores & Ardisson, 2015) (Table 1). The *k* parameter varied from 0.2 to 0.7 year^−1^ and the *t*_0_ parameter ranged between -0.8 and -0.4 years. A total of 1,000 repeated samplings were conducted to generate the *k* and *t*_0_ parameters, assuming a uniform, non-informative distribution (U). Finally, *M*_*t*_ values were estimated from the following equations:

(13) \begin{eqnarray*}k& \approx \mathrm{U}({k}_{min}.{k}_{max})\end{eqnarray*}

(14) \begin{eqnarray*}{t}_{0}& \approx \mathrm{U} \left[ { \left( {t}_{0} \right) }_{min}.{ \left( {t}_{0} \right) }_{min} \right] \end{eqnarray*}

### Statistical comparison of Natural mortality between scenarios

Generalized Linear Models (GLM) were applied to know the statistical effect of age-at-maturity scenarios on *M* for GIM and the Chen & Watanabe (1989) methods. *M* was the response variable while the explanatory variables were the maturity scenarios, the gnomonic stages and the ages. An interaction between gnomonic stages (for GIM), age (for Chen y Watanabe method) and stages of maturity was carried out assuming an identity link function and Gaussian error distribution (*η* = *μ*) in order to know if there is a difference between and stages with respect to the maturity scenarios (Nelder & Baker, 1972).

## Results

### Natural mortality coefficients estimated by the gnomonic intervals model

The estimates of the *M* coefficient derived using the gnomonic intervals model included the highest values of natural mortality during the larval phase covered by the first four gnomonic intervals (Table 2). A considerable reduction in *M* values was observed in the juvenile and adult phases (Table 2). The *M* estimates varied depending on the different scenarios of age-at-maturity considered, such that magnitude of the coefficients in the adult stage was higher in late maturity scenario, whereas in the juvenile stage the magnitude of *M* decreased (inverse effect) (Table 2). The daily survival rate from one gnomonic interval to another interval is shown in Table 2.

### Natural Mortality Coefficients estimated by the Chen & Watanabe (1989) Model

The *M* estimates obtained using the Chen & Watanabe (1989) model suggested that there was considerable variation among ages. It was generally observed that in the late maturity scenario the (Chen & Watanabe, 1989) model tends to estimate lower natural mortality values (Table 3). The variability of the estimates obtained for the older ages (>5 years) decreased in the late maturity scenario (Table 3).

### Statistical comparison of Natural mortality between scenarios

The differences in *M* among stages were highly significant for both the gnomonic interval method and the Chen & Watanabe method. However, for the GIM method, the maturity scenario was not significant, nor was the interaction between scenarios and stages. This suggests that the age-at-maturity applied by both scenarios (1.5 and five years) does not have a significant effect on natural mortality. For the method of Chen & Watanabe it was found that, both maturity scenarios, the age and the interaction between both variables showed an incidence on the values of natural mortality where the mortality values were lower when the age and the age at maturity are more advanced (Table 4).

**Table 2:** Natural mortality estimates in the two different scenarios proposed by the Gnomonic Interval Method (GIM) routine. Minimum and maximum values are referred to the interquartile limits of the total estimates for each stage in each of the scenarios. The first column express the age at which the first sexual maturity age is reached, that corresponds to each of the proposed scenarios.

		Stage of development											
Age at maturity (scenarios)		Early auricularia (year^−1^)	Survival (day^−1^)	Mid auricularia (year^−1^)	Survival (day^−1^)	Late auricularia (year^−1^)	Survival (day^−1^)	Doliolaria + pentactula (year^−1^)	Survival (day^−1^)	Juvenile (year^−1^)	Survival (day^−1^)	Adult (year^−1^)	Survival (day^−1^)
Early maturity (1.5 years)	Median	626.48	17.97	417.65	31.84	208.83	56.43	113.91	73.19	2.29	99.37	0.41	99.88
	Min	591.69	19.76	394.46	33.93	197.23	58.25	107.58	74.47	2.16	99.40	0.39	99.89
	Max	645.63	17.05	430.42	30.75	215.21	55.453	117.39	72.49	2.36	99.35	0.43	99.88
Late maturity (5 years)	Median	627.39	17.92	418.26	31.79	209.13	56.38	114.07	73.16	0.69	99.811	0.72	99.80
	Min	595.59	19.55	397.06	33.69	198.53	58.04	108.29	74.32	0.65	99.82	0.68	99.81
	Max	646.66	17.00	431.1	30.69	215.55	55.40	117.57	72.46	0.71	99.80	0.74	99.79

**Table 3:** Natural Mortality estimates of the Chen & Watanabe (1989) model, for the four different proposed scenarios. Natural Mortality (*M*) median was estimated for each age of the organism. It is presented in the interquartile range that contains the 50% of the estimates.

	Early maturity (1.5 years)			Late maturity (5 years)		
Relative age (years)	Median (year^−1^)	Lower 25% (year^−1^)	Upper 75% (year^−1^)	Median (year^−1^)	Lower 25% (year^−1^)	Upper 75% (year^−1^)
0	1.91	1.67	2.23	1.92	1.67	2.23
1	0.92	0.85	1.00	0.92	0.85	1.00
2	0.81	0.73	0.90	0.66	0.57	0.74
3	0.91	0.84	0.97	0.56	0.47	0.66
4	0.92	0.89	0.96	0.52	0.43	0.62
5	0.86	0.83	0.90	0.49	0.39	0.60
6	0.72	0.59	0.85	0.52	0.43	0.62
7	0.51	0.27	0.75	0.53	0.46	0.62
8	0.49	0.24	0.72	0.53	0.47	0.61
9	0.46	0.23	0.66	0.50	0.46	0.58
10	0.41	0.21	0.59	0.47	0.44	0.55

**Table 4:** Statistical differences between scenarios by Gnomonic interval model (Caddy, 1996) and Chen & Watanabe (1989) model. Stages: Early Auricularia, Mid Auricularia, Late Auricularia, Doliolaria + Pentactula, Juvenile and Adult. Gnomonic interval model with 91% of deviance explained; Chen & Watanabe model with 46% of deviance explained.

Model	Coefficients	Estimate	Std. Error	*t* value	Pr(>|*t*|)
Gnomonic Interval (GIM)	(Intercept)	660.150	2.958	223.206	<2e−16^***^
	Maturity	0.497	0.801	0.621	0.535
	Stages	−124.838	0.759	−164.383	<2e−16^***^
	Maturity:Stages	−0.114	0.206	−0.557	0.578
Chen & Watanabe	Intercept	1.377	0.006	213.70	<2e−16^***^
	Maturity	−0.208	0.009	−23.15	<2e−16^***^
	Age	−0.118	0.001	−89.65	<2e−16^***^
	Maturity:Age	0.020	0.001	11.25	<2e−16^***^

**Notes.**

Significance code: ‘^∗∗∗^’ 0.001.

## Discussion

There are more than 20 different methods to estimate mortality *M* in fish and invertebrates. Most of the methods are proposed for limited-data due to the difficulty in making accurate estimates of mortality since it is necessary to consider a number of factors that may influence in this estimation (e.g., depredation, competition, diseases, changes in the environment, among others). Most of these methods assume that *M* is constant in all phases of the life cycle in which fishing is intervening (Kenchington, 2014). There are few methods that consider estimations of *M* at different ages or stages during the life cycle of the organisms because it is necessary to have more information about the different stages of the life cycle of the species to achieve these estimates. Empirical relations of *M* as the proposes by Rikhter & Efanov (1976), Pauly (1980) and Jensen (1997) are good and quick estimates that allow timely inferences to be made especially when there are selective fisheries for some components of the population structure (e.g., adults). In the present study, although both methods (Chen & Watanabe and GIM) yielded values of *M* consistent with the estimates for other species of sea cucumbers (Table 5), it is important to point out that these estimates must be validated through direct methods in future research (field information). In the meantime the current estimates will allow to carry out stock assessment considering age as a source of variation of *M*. The fishing of *Isostichopus badionotus* is not selective because it is carried out through semi-autonomous diving and the catch is usually made up of juveniles and adults which *M* estimates by gnomonic phases or the Chen & Watanabe method should be considered in future stock assessments. In the case of the GIM method, it has the advantage of being originally developed for short-lived invertebrates (Caddy, 1996) and later adapted for species with greater longevity (Martínez-Aguilar, Arreguín-Sánchez & Morales-Bojórquez, 2005). Also, it is possible to take advantage of the information of the species in which the duration of each one of the phases of its life cycle has already been determined, either by direct observations or through aquaculture e.g., *I. badionotus* determined by Zacarías-Soto et al. (2013) who found consistencies in the duration of the gnomonic phases from spawners from the natural environment.

**Table 5:** Natural mortality coefficients reported for adults of different tropical and subtropical species of sea cucumbers by region.

Species	Natural Mortality (year^−1^)	Method	Region	Source
*Holothuria atra*	1.02	k	Islas Marshall Micronesia (T)	Ebert (1978)
*Holothuria floridana*	0.72	a	Q. Roo, Mexico (T)	De la Fuente-Betancourt et al. (2001)
*Isostichopus fuscus*	0.51	b	Baja California, Mexico (T-T)	Herrero-Pérezrul et al. (1999)
*Isostichopus fuscus*	0.67 0.354	b, c, d, e, f, g	Baja California, Mexico (T-T)	Reyes-Bonilla & Herrero-Pérezrul (2003)
*Isostichopus fuscus*	0.40 0.31	h, e, g, i, j	Baja California, Mexico (T-T)	Glockner-Fagetti, Calderon-Aguilera & Herrero-Pérezrul (2016)
*Thelenota ananas*	0.50-0.63	f	Nueva Caledonia (T)	Conand (1988)
*Stichopus chloronotus*	1.79	f	Nueva Caledonia (T)	Conand (1988)
*Actinopyga echinites*	0.64	f	Nueva Caledonia (T)	Conand (1989)
*Actinopyga mauritania*	1.45	f	Nueva Caledonia (T)	Conand (1989)
*Isostichopus badionotus*	0.41 0.62	l, m		Present study

**Notes.**

(T) Tropical (T-T) Tropical Temperate a Beverton & Holt (1959) b Pauly (1980) c Alagaraja (1984) d Chávez (1995) e Jensen (1997) f Rikhter & Efanov (1976) g Hewitt & Hoenig (2005) h Dunn, Francis & Doonan (2002) i Taylor (1959) j Gayanilo Jr & Pauly (1997) k Van Sickle (1977) l Caddy (1996) m Chen & Watanabe (1989)

The intensity of exploitation of *Isostichopus badionotus* in the Eastern Atlantic, Caribbean Sea and Gulf of Mexico by Cuba, Nicaragua, Venezuela, Puerto Rico, Panama, Mexico and Colombia (Buitrago & Boada, 1996; Guzmán & Guevara, 2002; Toral-Granda, Lovatelli & Vasconcellos, 2008; Tagliafico, Rangel & Rago, 2011; Ortega & Alfonso, 2012; Purcell et al., 2013; Poot-Salazar, Hernández-Flores & Ardisson, 2015; Narváez-Serrano, 2016) is a major concern among fishery managers in these countries. This has stimulated additional studies to better understand the biological and ecological behavior of this species and as a result, to improve the management plans for the resource.

In general, natural mortality estimates for different species of sea cucumbers are scarce. Some estimates have been obtained using a wide variety of methods for different species (Table 5), and all the studies assume a constant *M* during exploitation of the fishery across life stages rather than estimating different *M* values for different ages or life stages. This important source of variation should be considered in the assessment of population parameters and stock evaluations (Gosselin & Qian, 1997; Johnson et al., 2014).

Natural mortality estimates in the present study lay within the interval of estimates obtained for the congeneric species *I. fuscus* that lives in the tropical Eastern Pacific (Herrero-Pérezrul et al., 1999; Reyes-Bonilla & Herrero-Pérezrul, 2003; Glockner-Fagetti, Calderon-Aguilera & Herrero-Pérezrul, 2016), as well as other tropical species such as *Thelenota ananas*, *Actinopyga echinites* and *Holothuria floridana* (Conand, 1988; De la Fuente-Betancourt et al., 2001) (Table 5). Other tropical species such as *Holothuria scabra* (Purcell & Simutoga, 2008), reach age-at-maturity (or length-at-maturity) relatively rapidly compared to temperate species such as *Cucumaria frondosa* (Hamel & Mercier, 1996). The late maturity scenario showed a decrease in the gnomonic estimates of natural mortality coefficients although these were not significantly different from those estimated for early maturity. Organisms that have a longer life must exhibit a decrease in natural mortality coefficients at later ages to meet the assumption of a population in a state of equilibrium.

Application of the GIM model allowed calculation of *M* estimates during the early stages of the life cycle and, as expected, yielded higher natural mortality coefficients, from 626.48 to 113.91 year^−1^, for the early auricularia to pentactula stages. These were the highest natural mortality coefficients obtained during the first 22 days of the life of *I. badionotus*. The Chen & Watanabe (1989) model provided annual estimates; for age-at-zero, that includes all the planktonic phases and a large part of the juvenile stage, the natural mortality was between 1.67 and 2.23 year^−1^. This reflects only the *M* of the juvenile stage because this model does not consider information about the larval phases of the species. Additionally, the Chen & Watanabe (1989) model requires parameters of the von Bertalanffy equation which does not consider a species’ planktonic phase.

Natural mortality coefficients calculated for the juvenile and adult phases demonstrated consistency between the two models. The GIM model also showed lower variability in the estimated coefficients than the Chen & Watanabe (1989) model. This occurs because the former model assumes a constant death probability within each gnomonic interval (development phase of the life cycle), whereas the natural mortality coefficients estimated from the Chen & Watanabe (1989) model represent the interquartile range of the age at zero and one year for the juvenile phase and from two to ten years for the adult phase. Both methods can yield adequate results and the use of the estimates will depend on the objectives of future studies (e.g., aquaculture or fisheries assessments). Estimates of *M* have high dependence with the growth and development of organisms. *M* can commonly be referred to three phases: initial, stable mortality and mortality by senescence and in turn these phases are linked to the three main growth phases: initial growth, stable growth and senescence (Chen & Watanabe, 1989). Therefore, by varying important life stages as the age-at-maturity will undoubtedly affect the estimates of natural mortality due to the different physiological processes that act in this phase as the energy investment to the development of the gonads more than the growth.

A very important aspect of stock assessment is the uncertainty of the population parameters used in the different models. Specifically, incorrect values for *M* may affect the fishing mortality coefficient (*F*) estimated from the total mortality coefficient (Z) ( *F* = *Z* − *M*), as well as the population abundance (Clark, 1999). In this study, the main sources of uncertainty were incorporated into the natural mortality coefficient estimates as a population parameter. Incorporation of uncertainty in the Chen & Watanabe (1989) model will not provide discrete estimates of *M* for a species, because several estimates of *k* and *t*_0_ parameters may be available for the species worldwide. It is thus better to generate a series of random estimates by considering the minimum and maximum values of these parameters that will ultimately provide improved *M* estimates, an important approach used in the current study.

Based on the available knowledge on the planktonic larvae of benthic marine invertebrates, the high mortality during the larval phases of *I. badionotus* may be attributable to problems with fertilization, inadequate food supply, lethal and sublethal temperatures, absence of an appropriate substrate for settlement, offshore transport by marine currents and predation (Rumrill, 1990). The juvenile phase immediately follows recruitment and settlement in the substrate, and is a critical phase during which the habitat and behavioral changes affect mortality. During this phase, there is a marked increase in size in sea cucumber species and extensive formation of dermal ossicules. Thus, in general, high natural mortality coefficients are reported for juveniles of benthic marine invertebrates (*sensu* Gosselin & Qian, 1997).

On the other hand, the change to the adult phase occurs when the organism reaches sexual maturity and is able to reproduce, a transition typically associated with important physiological changes. The lowest natural mortality coefficients were observed in the present study during this phase, in agreement with results of (Post & Evans, 1989), who observed that in fish, larger organisms are better able to survive adverse conditions.

## Conclusions

Estimates of the natural mortality coefficient (*M*) generated in this study may provide basic input in stock assessment models that are a prerequisite for the adequate management of fished populations. However, these estimates should be validated in future research by direct methods that consider the factors that could influence the estimates of *M* (depredation, competition, environment, diseases, among others). Consideration of *M* as a variable instead of a constant parameter in stock assessment might contribute to more robust predictions and preclude greater biases in the estimate of population sizes, for example using Virtual Population Analysis (Pope, 1972).

Estimates of natural mortality for the sea cucumber (*I. badionotus*) are relevant to favor adequate assessments of their populations, which are mostly in phases of overexploitation fisheries.

##  Supplemental Information

10.7717/peerj.5235/supp-1 Supplemental Information 1 These results were obtained from GIM model software Raw data (simulated data, Fig. 2).

10.7717/peerj.5235/supp-2 Supplemental Information 2 R code for simulation of Chen & Watanabe model

## Additional Information and Declarations

### Data Availability

The following information was supplied regarding data availability: The data and code are provided as Supplemental Files.
